# Combination therapy of brain radiotherapy and EGFR-TKIs is more effective than TKIs alone for EGFR-mutant lung adenocarcinoma patients with asymptomatic brain metastasis

**DOI:** 10.1186/s12885-019-6005-6

**Published:** 2019-08-09

**Authors:** Yanxin Chen, Jianping Wei, Jing Cai, Anwen Liu

**Affiliations:** 1 Department of oncology, The second affiliated hospital of Nanchang University, Jiangxi province, Nanchang, 330006 China; 2 Jiangxi key laboratory of clinical translational cancer research, The second affiliated hospital of Nanchang University, Jiangxi province, Nanchang, 330006 China

**Keywords:** EGFR-TKIs, Lung adenocarcinoma, Brain metastasis, Radiotherapy

## Abstract

**Background:**

The treatment strategy for brain metastasis (BM) in patients with epidermal growth factor receptor (EGFR) -mutant lung adenocarcinoma (LAC) remains controversial. In the present study, we compared the efficacy of brain radiotherapy (RT) in combination with tyrosine kinase inhibitors (TKIs) and TKIs alone for advanced LAC patients with EGFR mutations and BM.

**Methods:**

We retrospectively studied 78 patients diagnosed with EGFR-mutant LAC who developed BM. These patients were divided into two groups: 49 patients in the combination treatment group who received brain RT in combination with EGFR-TKIs (including 23 patients with asymptomatic BM before RT); 29 patients in the TKI group who received EGFR-TKI targeted therapy alone (including 22 patients with asymptomatic BM before TKI treatment).

**Results:**

The median intracranial progression-free survival (iPFS) of the combination treatment group was longer than that of the TKI alone group (21.5 vs. 15 months; *P* = 0.036). However, there were no significant differences in median progression-free survival (PFS, 12 vs. 13 months; *P* = 0.242) and median overall survival (mOS, 36 vs. 23 months; *P* = 0.363) between the two groups. Further analysis of asymptomatic BM showed that both the median iPFS and the mOS of the combination treatment group were significantly longer than for the TKI alone group (iPFS, 21.5 vs. 14.8 months, *P* = 0.026; mOS, 36 vs. 23 months, *P* = 0.041). Cox multivariate regression analysis found no independent adverse predictors of iPFS in all patients.

**Conclusions:**

The synchronous combination of brain RT and TKIs was superior to EGFR-TKIs alone for EGFR-mutant LAC patients with BM. The combination treatment group exhibited longer iPFS, while the PFS and OS were not significantly different between the two groups. In addition, the combination treatment could result in better iPFS and OS in those with asymptomatic BM. Therefore, addition of brain RT was useful for intracranial metastatic lesions.

## Background

An estimated 18.1 million new cases of cancer and 9.6 million cancer-related deaths occurred as of 2018 [1]. Lung cancer is the most commonly diagnosed cancer (11.6%) and also the leading cause of cancer-related death (18.4% of all cancer deaths) [1]. During the course of the disease, 22–54% of non-small-cell lung carcinoma (NSCLC) patients develop brain metastasis (BM) [2] [3]. Studies have shown that the incidence of BM in lung adenocarcinoma (LAC) is higher than that in other subtypes of NSCLC. About 45–52% of LAC patients develop BM during the course of the disease [4]. BM is a common complication in LAC patients and an important cause of morbidity and mortality [5]. In general, the prognosis of patients with BM still remains poor. The epidermal growth factor receptor (EGFR) gene plays a critical role in regulating normal cell proliferation, apoptosis, and other cellular roles [6, 7]. Studies have shown that EGFR mutation is significantly associated with the risk of BM after initial diagnosis and radical resection of LAC [8]. Patients with EGFR mutations are more vulnerable to BM than those with wild-type EGFR. At initial diagnosis [9], BM is found in approximately 25% of patients with EGFR mutations. Therefore, it is urgently necessary to develop reasonable and effective treatments to address this.

The development of radiotherapy (RT) and targeted therapy, and particularly, the combination of RT and targeted therapy, in recent years, has greatly prolonged the median overall survival (OS) and median progression-free survival (PFS) for NSCLC patients with BM [10]. For EGFR-mutant NSCLC patients with BM, tyrosine kinase inhibitors (TKIs) can effectively control intracranial position of the disease [11]. Brain RT can also effectively control intracranial lesions [12]. Based on the advantages of these individual treatments, we posited that a combination therapy might be effective. However, based on currently available data, the efficacy of such a combination remains controversial. Some studies have shown that brain RT in combination with EGFR-TKIs is more effective than TKIs alone [13]. However, other studies have shown that TKIs in combination with RT has no beneficial effects on intracranial PFS (iPFS) or OS [14].

Furthermore, patients with BM but no intracranial symptoms do not require immediate relief, and suitable treatment options are still disputed. EGFR-TKIs have been used for the treatment of asymptomatic BM. However, only a few studies have assessed the effects of EGFR-TKIs in combination with RT. In the present study, we aimed to explore whether combination therapy of TKIs and RT could benefit asymptomatic BM.

We retrospectively evaluated the efficacy of combination therapy and TKIs alone in the treatment of LAC patients with BM and EGFR mutations. We also evaluated the efficacy of these two therapeutic regimens in asymptomatic BM.

## Methods

### Patients

A total of 391 patients were diagnosed with LAC between April 2014 and June 2018 at the Second Affiliated Hospital of Nanchang University, of which 78 patients were diagnosed with stage IV LAC, and these patients were also detected with EGFR mutation and BM. These 78 patients with BM at preliminary diagnosis were retrospectively enrolled and analyzed in the present study. The inclusion criteria were set as follows: 1) LAC diagnosis by percutaneous lung biopsy or fiberoptic bronchoscopy; or reconfirmation of a pathological section as LAC after consultation in our hospital, followed by EGFR mutation diagnosis by genetic test; 2) older than 18 years old; 3) BM diagnosis by craniocerebral magnetic resonance imaging (MRI); 4) type of comparison: TKIs alone or combination of brain RT and TKIs. The exclusion criteria were set as follows: 1) patients who developed BM after taking EGFR-TKIs; 2) patients who did not receive EGFR-TKIs after stereotactic radiosurgery (SRS) or whole brain radiotherapy (WBRT); and 3) patients who received TKIs before or after brain RT.

Clinical information of patients was collected, including age, gender, smoking status, EGFR mutation status, number of BM, extracranial metastasis, EGFR-TKI drugs, type of brain RT, an update of the Graded Prognostic Assessment for Lung Cancer using Molecular Markers (lung-molGPA), Karnofsky Performance Status (KPS) score, and the location of the primary disease. Importantly, the absence or presence of intracranial symptoms here refers to the beginning of treatment, rather than the entire course of disease progression. Asymptomatic BM was defined as no increased intracranial pressure, dizziness, headache, nausea or vomiting, visual impairment, mental symptoms, and seizures or signs of focal neurological symptoms, regardless of whether there are symptomatic in other parts, including the lungs. Age, number of BM, extracranial metastasis, and lung-mol GPA scores reflected the current status of all patients who received treatment. The type of EGFR mutation was divided into the common EGFR mutations: exon 19 deletion (19del) and Leu858Arg point mutation (L858R). Rare EGFR mutations were defined as those other than 19del and L858R. Primary intracranial disease progression means that other systemic lesions were stable, while intracranial lesions progressed. A total of 78 patients were treated with EGFR-TKIs (gefitinib 250 mg qd; erlotinib 150 mg qd; icotinib 125 mg, tid). For the brain radiation group, the Elekta Versa HD medical linear accelerator and the Monaco planning system were used. The total radiation dose for WBRT was 30 Gy administered in 10 fractions (once a day, 5 days per week, 3 Gy each time). The total dose for SRS was 25 Gy administered in 5 fractions (once a day, 5 days per week, 5 Gy each time), 30 Gy administered in 5 fractions (once a day, 5 days per week, 6 Gy each time), or 35 Gy administered in 5 fractions (once a day, 5 days per week, 7 Gy each time). Each patient underwent laboratory and imaging examinations, including CT scans of the chest and upper abdomen, computed tomography (ECT) of the bone, and MRI of the brain. Patients were evaluated for efficacy 1 month after the end of treatment, followed by 2 months and then every 3 months. The therapeutic effect was evaluated by brain MRI, chest CT and upper abdominal CT. Tumor response was assessed by the Response Evaluation Criteria in Solid Tumors (RECIST) 1.1.

### Statistical analysis

The iPFS was defined as the time from the initiation of RT in combination with EGFR-TKIs or TKIs alone to the time of intracranial progression or death without documented progression, the last follow-up time for patients who did not progress or died was a censored value. PFS was defined as the time from the onset of treatment to any disease progression in the body or death without documented progression, the last follow-up time for patients who did not progress or died was a censored value. OS was defined as the time from the initiation of RT in combination with EGFR-TKIs or TKIs alone to death or last follow-up if they were still alive. Survival analysis was performed using Kaplan-Meier curves. The effects of potential variables on PFS were assessed by univariate analysis. Multivariate testing was performed by Cox regression analysis. Statistical analysis was performed by using SPSS software version 22.0.

## Results

### Patients’ characteristics

**Fig. 1:**
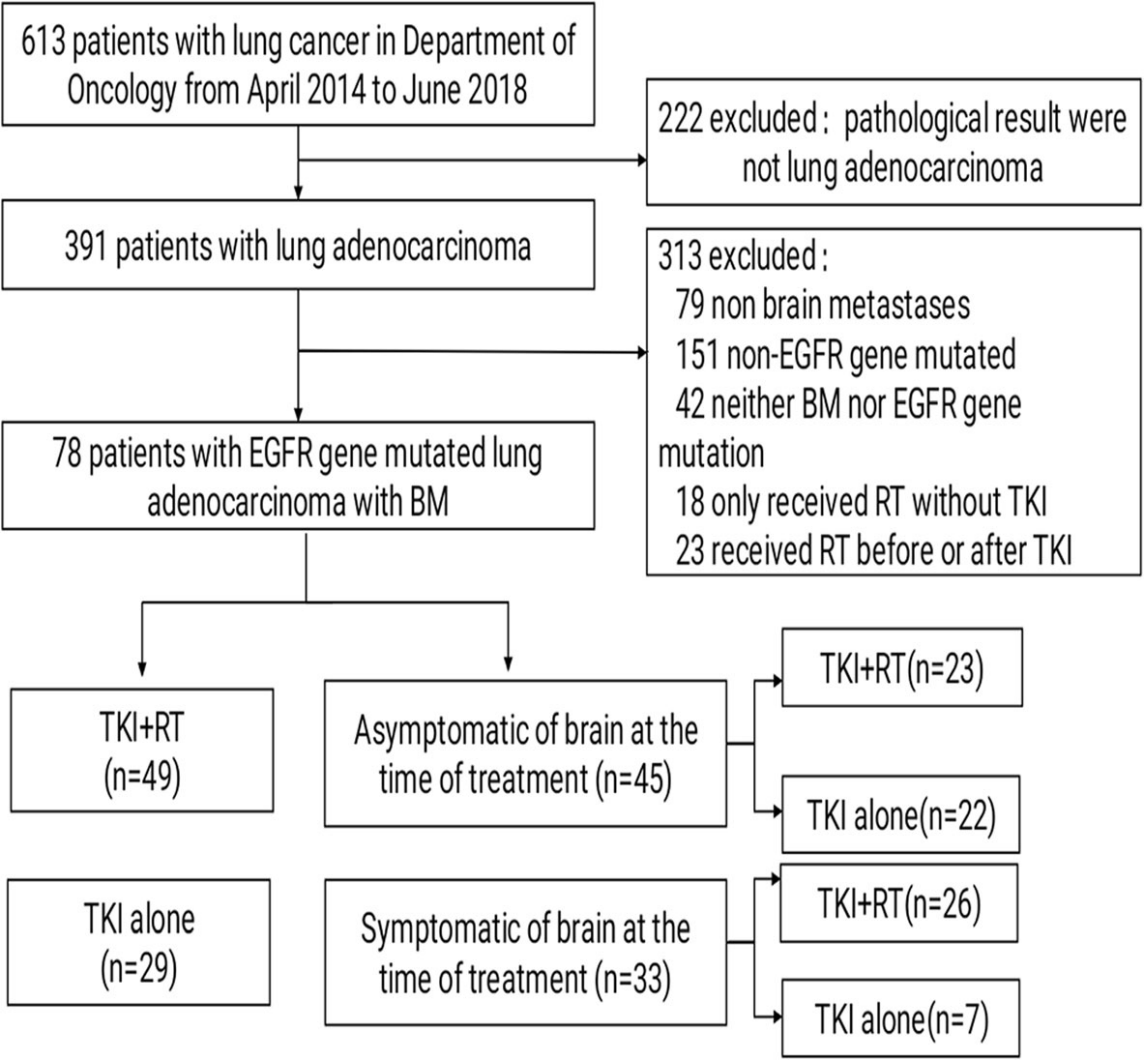
The flow chart of the patient queue

We included 613 patients who were diagnosed with LAC from April 2014 to June 2018 at the Second Affiliated Hospital of Nanchang University. Among them, 391 LAC patients were selected according to the inclusion criteria. Finally, 78 LAC patients diagnosed with EGFR mutations who developed BM were enrolled in the present study (Fig. 1). Table 1 shows the baseline characteristics of patients. Among them, 49 (62.8%) received a combination therapy of brain RT and EGFR-TKIs, and the other 29 (37.1%) received EGFR-TKI targeted therapy alone. Our data showed that 45 patients (57.7%) had asymptomatic BM at the beginning of treatment, of which, 22 patients were treated with TKIs alone and 23 patients received the combination therapy of TKIs and RT. Table 2 shows the baseline characteristics of these patients.

**Table 1:** Clinical and Molecular Characteristics of Included Patients

	TKI alone		TKI + RT		
Characteristic	(*n* = 29)	%	(*n* = 49)	%	*p* Value
Age (years)					0.639
Median	59		59		
Range	32–74		35–83		
< 65	21	72.4	33	67.3	
≥65	8	27.6	16	32.7	
Gender					0.729
Male	13	44.8	20	40.8	
Female	16	55.2	29	59.2	
Smoking history					0.729
Never or light	16	55.2	29	59.2	
Heavy	13	44.8	20	40.8	
EGFR mutation					0.323
Del19	9	31.0	22	44.9	
L858r	18	62.0	26	53.1	
Other	2	7.0	1	2.0	
BM no. at time of diagnosis					0.292
≤3	16	55.2	21	42.9	
>3	13	44.8	28	57.1	
Extracranial metastases					0.454
Yes	24	82.8	37	75.5	
No	5	17.2	12	24.5	
Intracranial Symptoms					0.012
Without	22	75.9	23	46.9	
With	7	24.1	26	53.1	
Lung-mol GPA classification					0.339
0–1	1	3.5	2	4.1	
1.5–2	9	31.0	12	24.5	
2.5–3	12	41.4	22	44.9	
3.5–4	7	24.1	13	26.5	
Primary tumor location					0.128
Left Lung	17	58.6	20	40.8	
Right Lung	12	41.4	29	59.2	
KPS score(%)					0.801
<80	6	20.7	9	18.4	
≥80	23	79.3	40	81.6

**Table 2:** Clinical and Molecular Characteristics of patients with asymptomatic brain metastases

	TKI + RT		TKI alone		
Characteristic	(*n* = 23)	%	(*n* = 22)	%	*p* Value
Age (years)					0.608
Median	61		59		
Range	44–75		41–74		
< 65	14	60.9	15	68.2	
≥65	9	39.1	7	31.8	
Gender					0.295
Male	10	43.5	13	59.1	
Female	13	56.5	9	40.9	
Smoking history					0.295
Never or light	13	56.5	9	40.9	
Heavy	10	43.5	13	59.1	
EGFR mutation					0.155
Del19	12	52.2	16	72.7	
L858r	11	47.8	6	27.3	
Other	0	0	0	0	
BM No. at time of diagnosis					0.463
≤3	14	60.9	11	50.0	
>3	9	39.1	11	50.0	
Extracranial metastases					0.477
Yes	18	78.3	19	86.4	
No	5	21.7	3	13.6	
Lung-mol GPA classification					0.266
0–1	0	0	0	0	
1.5–2	3	13.1	6	27.3	
2.5–3	13	56.5	10	45.4	
3.5–4	7	30.4	6	27.3	

The final follow-up date of the study was October 29, 2018. At the time of last follow-up, 28 patients survived without signs of disease progression, 21 patients exhibited signs of disease progression, 27 patients had died of disease progression, one patient had died of unrelated causes, and one patient was lost during follow-up.

In the present study, 49 of 78 patients received combination therapy of RT and TKIs (WBRT in 35 and SBRT in 14), and 29 patients received TKIs alone. Intracranial progression was detected in 31 of the 78 patients (39.7%). Intracranial progression occurred in 41.8% (12 out of 29) of the patients who received EGFR-TKIs alone, compared with 38.7% (19 out of 49) for patients who received combination therapy of EGFR-TKIs and brain RT. Primary intracranial disease progression was noted in 27.6% (8 out of 29) of patients who received TKIs alone, compared to 18.3% (9 out of 49) in patients receiving combination therapy.

### Survival outcomes

**Fig. 2:**
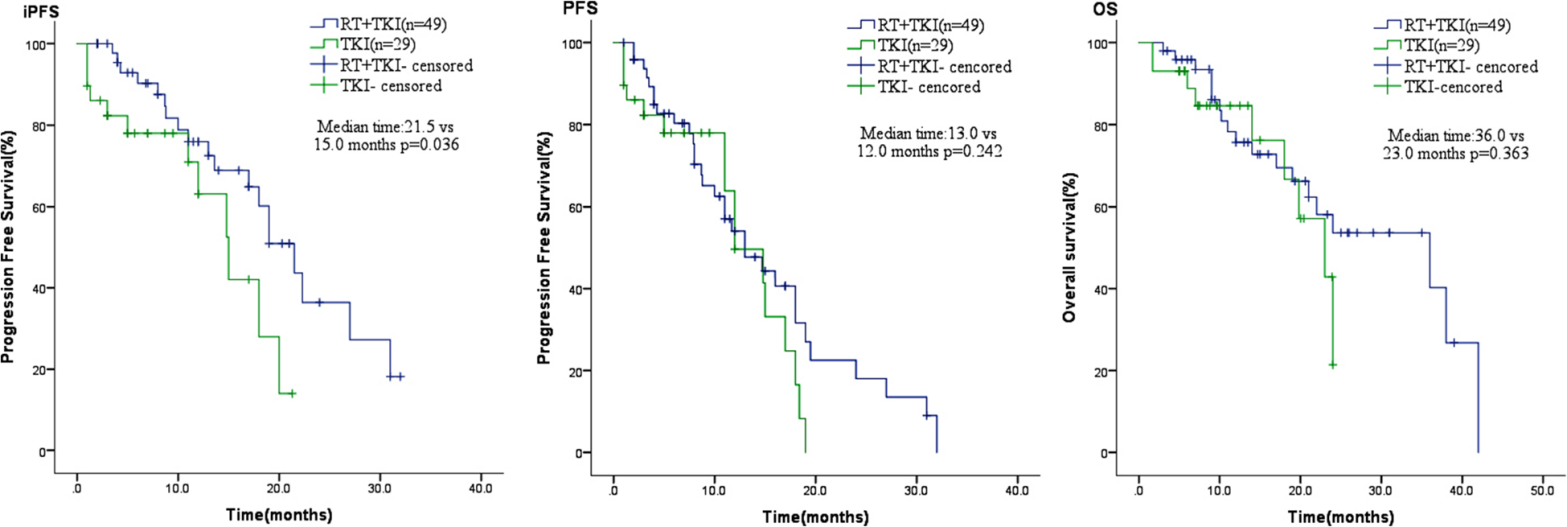
Combination therapy group had similar PFS and OS, but better iPFS than only TKIs therapy group in LAC patients with EGFR-mutant and BM

The median PFS of the study population was 11 months. The median iPFS of patients receiving RT + TKIs was 21.5 months, which was significantly longer than that of those receiving EGFR-TKIs alone (median iPFS, 15 months, *P* = 0.036). However, the median PFS (mPFS, 12 months versus 13 months; *P* = 0.242) and mOS (36 months versus 23 months, *P* = 0.363) were not significant different in these two groups, although the mPFS and mOS in the combination treatment group were higher (Fig. 2).

**Fig. 3:**
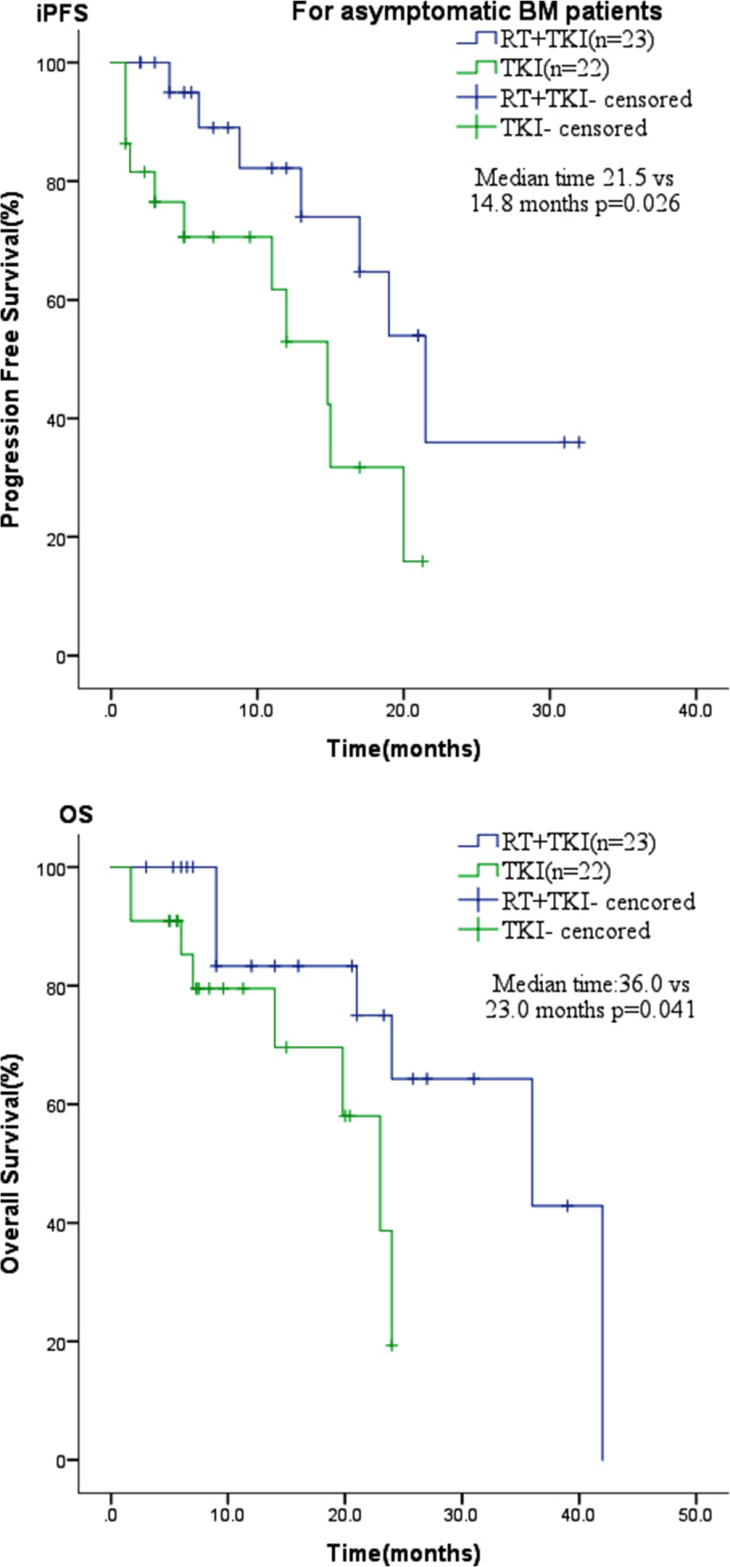
For asymptomatic BM patients, the iPFS and OS in combination therapy group were longer than in the TKIs alone group

For patients with asymptomatic BM, the median iPFS was 21.5 months for patients who received RT + TKIs (*n* = 23) and 14.8 months for patients who received EGFR-TKIs alone (*n* = 22, *P* = 0.026). The OS was prolonged in patients who received RT + TKIs (36 months, *P* = 0.041) compared with those who received TKIs alone (23 months) (Fig. 3).

Cox multivariate regression analysis found no independent adverse predictors of iPFS in all patients (Table 3).

**Table 3:** Multivariate analysis of prognostic factors for iPFS in 78 patients

	*P*	HR	95.0% CI for HR	
			Lower	Upper
Gender (male vs. female)	0.137	0.16	0.02	1.78
Age (<65y vs. ≥65y)	0.890	0.94	0.37	2.34
Smoking history (never vs. smoking)	0.134	5.57	0.59	52.53
EGFR mutation (L858r vs. Del19 vs. rare mutations)	0.665	0.83	0.36	1.91
BM No. (≤3 vs.>3)	0.315	0.63	0.26	1.55
Metastases(B vs. B + E)	0.740	0.82	0.26	2.58
Intracranial symptom (have vs. No)	0.267	0.60	0.24	1.48
First-line treatment (Yes vs. No)	0.445	1.55	0.51	4.74
KPS score (<80% vs ≥80%)	0.918	0.94	0.26	3.37

*No*. Number, *B* brain, *B + E* brain and extracranial metastasis

## Discussion

Based on our small-scale retrospective study, we could conclude the following: 1) a combination therapy of RT and TKIs could improve iPFS, while OS and PFS were not significantly prolonged compared with TKIs alone; 2) for patients with asymptomatic BM, the iPFS and OS of the combination therapy group were longer compared with the TKIs alone group.

Multiple retrospective studies have reported similar results [13, 15–17]. For example, a systematic review and meta-analysis consisting of 12 studies found that in EGFR-mutant NSCLC patients who develop BM, cranial RT followed by TKIs improved iPFS compared with upfront TKI, showing that the use of upfront EGFR-TKIs and delay of RT were associated with poor PFS [17]. Several mechanisms can explain the combined effect of TKI + WBRT on BM of NSCLC patients with EGFR mutation. Firstly, EGFR-TKI can inhibit the proliferation of tumor cells, inhibit the apoptosis pathway, and suppress DNA repair capability, making tumor cells more sensitive to RT [18, 19]. Secondly, RT can increase the effective concentration of TKIs by enhancing the blood brain barrier (BBB) permeability [20]. Finally, radiation can reduce the probability of the T790 M mutation [21, 22].

Magnuson et al. conducted a multi-institutional analysis consisting of 351 EGFR-mutant NSCLC patients who developed BM. The patients were divided into three groups: SRS followed by EGFR-TKI, WBRT followed by EGFR-TKI, or EGFR-TKI followed by SRS or WBRT. This analysis, however, demonstrated that the iPFS of these three groups was similar at 23 months, 24 months, and 17 months, respectively [16]. Byeon et al. have also shown that there are no differences between these treatments [23], although, their study used sequential cranial RT, in contrast to a synchronous combination of brain RT and TKIs in our study. Another study showed that based on the radiosensitizing effect, the duration of opening the BBB, and the reproductive death, it is reasonable to administer TKIs either concurrently or one week before RT [24]. Moreover, in a study by Yang et al., with 85 patients in the icotinib group and 91 patients in the WBRT group, the median iPFS of NSCLC patients with EGFR mutation and BM were 4.8 months and 10.0 months (*P* < 0.05), while the median OS were 20.5 months and 18.0 months (*P* > 0.05). Therefore, TKIs alone may be insufficient to treat BM of NSCLC [25].

Treatment strategies remain uncertain for patients with asymptomatic BM. In a study by Chen et al., combination RT showed no significant changes in intracranial TTP (*P* = 0.193) for asymptomatic patients [26]. Liu et al. reported that first-line treatment using brain RT fails to lengthen the survival time of patients with EGFR mutation and asymptomatic BM [27]. Based on the high intracranial response rates, TKIs alone have been proposed as initial treatment in patients with EGFR mutations and asymptomatic BM [28]. However, this approach can be associated with a higher risk of subsequent intracranial relapse. The use of primary TKIs can ameliorate the adverse effects of RT; however, it is unlikely to they can completely abrogate the need for subsequent RT. In addition, asymptomatic patients may have lower tumor load, stronger physical condition, and less systemic metastasis. Therefore, effective control of intracranial lesions is more meaningful for long-term survival of patients. For EGFR-mutant LAC patients with BM, cranial RT in combination with TKIs is a possible strategy, that may improve PFS and OS compared with TKIs alone. Wang et al. have also reported similar results, that delayed brain RT may lead to lower iPFS in NSCLC patients with EGFR mutation and asymptomatic BM (0.032) [29].

The TKIs used in our study included gefitinib, erlotinib, and icotinib. However, osimertinib has demonstrated a greater penetrating capacity of the mouse BBB compared with gefitinib, rociletinib, and afatinib, and could achieve sustained tumor regression in an EGFR mutated PC9 mouse model of BM [30]. Studies also found that osimertinib combined with RT could significantly reduce the proliferation of NSCLC cells harboring T790 M/L858R mutations in vitro and in vivo, reduce cell cycle arrest in G2/M phase, and could block RT-induced DNA double-strand breaks (DSB) repair, demonstrating its role in radiosensitivity [31]. A double-blind, phase III trial found that the frequency of central nervous system progression was lower in the osimertinib group compared with the standard EGFR-TKI group [32]. Two randomized phase II trials of Osimertinib with or without SRS for the treatment of EGFR mutant NSCLC with BM (NCT03497767 and NCT03769103) are about to begin, and we are looking forward to their results. In conclusion, osimertinib in combination with cranial RT may have a greater benefit in LAC patients with BM and EGFR mutation, and further studies are needed to assess its efficacy.

Our current study has certain limitations: (i) we only included patients from a single institution, and the patient population was, thus, relatively small; (ii) due to the retrospective nature of our study, undefined biases and/or confounding factors may have influenced clinical outcomes.

## Conclusions

Collectively, compared with EGFR-TKI treatment alone, combination therapy of TKIs and RT could significantly prolong iPFS. For patients with asymptomatic BM, the combination therapy showed beneficial effects on iPFS and OS, highlighting the usefulness of RT. Although combination therapy has grown in popularity in recent years, more prospective studies are needed to analyze different populations in order to achieve effective treatment.

## Data Availability

The datasets used and/or analyzed during the current study are available from the corresponding author on reasonable request.

## Abbreviations

BM: Brain metastases
EGFR: Epidermal growth factor receptor
KPS: Karnofsky performance status
LAC: Lung adenocarcinoma
lung-molGPA: An update of the graded prognostic assessment for lung cancer using molecular markers
NSCLC: Non-small cell lung cancers
OS: Overall survival
PFS: Progression free survival
RT: Radiotherapy;
SRS: Stereotactic radiosurgery
TKI: Tyrosine kinase inhibitors
WBRT: Whole brain radiotherapy
